# Factors associated with having less than 20 natural teeth in rural adults: a cross-sectional study

**DOI:** 10.1186/s12903-015-0147-y

**Published:** 2015-12-11

**Authors:** Su-Jen Tsai, Ming-Shyan Lin, Wen-Nan Chiu, Su-Whi Jane, Liang-Tse Tu, Mei-Yen Chen

**Affiliations:** 1 College of Nursing, Chang Gung University of Science and Technology, Taoyuan city, Taiwan, Republic of China; 2 Department of Internal Medicine, Chang Gung Memorial Hospital, Yunlin County, Taiwan, Republic of China; 3 Nursing Department, Chang Gung University of Science and Technology, Taoyuan city, Taiwan, Republic of China; 4 Division of Dentistry, Chang Gung Memorial Hospital, Yunlin County, Taiwan, Republic of China

## Abstract

**Background:**

Some systematic reviews have consistently indicated a positive link between Metabolic syndrome, impairedfasting glucose, all-cause or circulatory disease-related mortality, general health, periodontitis, and toothloss. This study was to examine the prevalence of number of remaining teeth <20 and associated risk factors among adults in a rural area of Taiwan.

**Methods:**

A community-based, cross-sectional study was conducted in southwestern coastal Taiwan in 2013; 6680 residents aged 20–64 years were studied. Oral hygiene, substance use, dietary habits, and metabolic syndrome were explored as potential risk factors for number of remaining teeth <20 using logistic regression analysis.

**Results:**

The mean number of remaining teeth was 24.6 (SD = 7.4), and 16.3 % (n = 1085) of the participants had number of remaining teeth <20. Men had significantly less frequent use of dental floss, unhealthy dietary habits, more substance use and metabolic syndrome than did women (*p* <0.001). However, women tended to have fewer teeth than men (*p* <0.001). After adjusting for potential confounders, older age (odds ratio [OR] = 4.56, 95 % confidence interval [CI]: 3.74–5.55), female (OR = 1.88, 95 % CI: 1.56–2.25), less education (OR = 2.40, 95 % CI: 1.90–3.02), infrequent use of dental floss (OR = 1.94, 95 % CI: 1.66–2.27), substance use (OR = 1.32, 95 % CI: 1.09–1.59), and number of metabolic syndrome components (OR = 1.10, 95 % CI: 1.04–1.16) were independently associated with a higher risk of number of remaining teeth <20.

**Conclusions:**

Number of remaining teeth <20 was highly prevalent among rural adults. In addition to unmodifiable factors, infrequent use of dental floss, substance use, and metabolic syndrome were risk factors associated with tooth loss.

## Background

Oral health is essential to overall health and quality of life, and it plays an important role in an individual’s capacity for biting, chewing, smiling, speaking, and psychosocial wellbeing [1]. A campaign, called “8020” aims for the elderly to retain at least 20 teeth at 80 years of age; this campaign was launched in Japan in 1991 [2]. Theoretically, most individuals should expect to maintain functional dentition throughout our lives. Some studies have indicated that physical indices and oral health conditions in all who achieved the 8020 goals were better than the non-achievers [2, 3]. Evidence supports the relationship between tooth loss and health outcomes, including malnutrition and mortality [4].

For many high-income countries, 5–10 % of public health expenditure relates to oral health [1]. Many studies have indicated that tooth loss is associated with metabolic syndrome (MetS) [5], increased risk of stroke, ischemic heart disease, cognitive impairment, and all-cause and cardiovascular mortality [6–10]. The literature also shows that risk factors associated with tooth loss in adults include age, female sex, lower educational level [11–13], poor oral hygiene [12, 14], high blood pressure, high fasting blood glucose (FBG), obesity [5, 15, 16], betel nut chewing, cigarette smoking [17, 18], diabetes mellitus [19–21], and unhealthy diet [1].

Some systematic reviews [22–24] have consistently indicated a positive link between MetS, impaired fasting glucose, all-cause or circulatory disease-related mortality, general health, periodontitis, and tooth loss. The mechanism might be due to chronic infections, which have been associated with increased levels of inflammatory cytokines and cardiovascular disease. For instance, in a prospective cohort study, individuals with *Helicobacter pylori* infection were 2.7 times more likely to develop diabetes than were seronegative individuals. Even after controlling for insulin resistance, C-reactive protein and interleukin-6 levels did not attenuate the effect of *H. pylori* infection [25].

According to some evidences, the main causes of tooth loss are periodontal disease and dental caries and are mostly preventable [22–27]. For instance, using fluoride, brushing the teeth often (at least twice a day), flossing the teeth daily, undergoing regular dental check-ups every 6 months, choosing a healthy diet, and keeping blood glucose under control are all emphasized [22, 23]. If all individuals followed these rules, it would not be difficult to reach the 8020 aims. However, many people do not practice these habits daily, especially in disadvantaged areas [1–3]. In addition, few studies had been initiated in Taiwan. Therefore, the aim of the present study was to provide the prevalence of remaining less than 20 teeth and to examine the associated risk factors for tooth loss among adults in a southwestern coastal rural area of Taiwan.

## Methods

### Design, sample, and setting

This is part of a longitudinal study on health promotion for community health development led by community nursing faculty members and was performed in cooperation with a local hospital in Yunlin County, Taiwan. The community-based survey and descriptive cross-sectional design were implemented from September 2012 to August 2013. Participants were selected by convenience sampling at 27 villages in the southwestern coastal areas. Considering the modifiable factors related to the prevention of tooth loss, this study selected those aged <65 years for data analyses. The inclusion criteria were (1) age between 20 and 64 years, (2) complete independence in managing daily lives, (3) ability to complete the questionnaires in Mandarin or Taiwanese dialects either by self-administration or at an interview, (4) ability to walk to the local corporate hospital, and (5) provision of a signed informed consent before enrollment in the study. Exclusion criteria were (1) serious mental problems, including those that needed a disability certificate or a diagnosis of dementia by a physician or community welfare organization, (2) diagnosis of either type 1 or type 2 diabetes, or (3) inability to walk to the local hospital for physical examination. Due to the nature of community-based census, there was no formal sample size determination in this study.

### Instruments

1.  *Demographic characteristics* included age, sex, and educational attainment (years of education received or school level graduated).
2.  *Oral hygiene*: According to one of our previous studies regarding factors related with the number of remaining teeth (NRT) among diabetes in a neighboring county, 74 % of 703 participants had fewer than 20 NRT [14]. Ironically, 88.3 % reported they brushed their teeth more than twice a day, and 70 % did not frequently use dental floss as a regular habit. This phenomenon might be due to a misunderstanding regarding what it means to “brush teeth”. Therefore, in the present study, oral hygiene was assessed using the question “In your daily life, how often do you use dental floss after tooth-brushing each day.” Answers were categorized as frequent (≥ once a day) or infrequent (never/sometimes).
3.  *NRT* was obtained by counting the natural and filled teeth in the oral cavity by the trained research assistant.
4.  *Substance use:* According to official reports from the national health survey, a high prevalence rate of alcohol drinking, betel nut chewing, and cigarette smoking were found in the western coastal area of Taiwan and associated with some significant cancers [3]. These three habits were defined as substance use in the present study and were measured according to the responses to the following three questions. (A) “Do you regularly drink alcohol?” Participants were classified as “less consumption” if they had never drank alcohol or had not drank alcohol for 1 year, or “regular consumption’ if they were currently drinking. (B) “Do you chew betel nuts?” Participants were classified as “less consumption” if they had never chewed betel nuts or no longer chewed or “regular consumption” if they were currently chewing one quid or more per day. (C) “Do you smoke cigarettes?” Participants were classified as “less consumption” if they rarely or never smoked or “regular consumption” if they currently smoked one or more cigarettes per day. In addition, any one of the above habits was classified as consumption and was recorded as substance use.
5.  *Healthy dietary habits* were based on reports from the World Health Organization [28] and the published literature [29, 30]. Healthy diets for adults should contain at least 400 g (five portions) of fruits and vegetables a day, nuts and whole grains, five groups of food, and enough water instead of soft drink. In this study, healthy dietary habits were determined according to the answers to the following questions. (A) Vegetable consumption habits: “Do you eat three portions of vegetables every day?” The answer was classified as “deficient” if they answered never or sometimes, and as “not deficient” if they usually had at least three portions or one and half bowl-sized portions of vegetables 5 days per week. (B) “How often do you have five groups of nutrition each day, e.g. meat, milk, rice, vegetable, fruit, and mineral food?” (C) “How often do you have 1500 mL or at least eight bowl-sized cups of water each day? The answer of B and C were classified as “deficient” if they answered never or sometimes, and as ‘not deficient’ if their answer was usually or always. Inadequate consumption of any one of the above was classified as a deficiency and was recorded as an unhealthy dietary habit.
6.  *Metabolic syndrome* was based on the national standard of the Ministry of Health and Welfare [29] and defined as abnormal values for three of the following five components: (a) FBG ≥ 100 mg/dL, (b) systolic and diastolic blood pressure ≥ 130/85 mm Hg); (c) HDL-C <40 mg/dL for men or <50 mg/dL for women, (d) TG ≥ 150 mg/dL; and (e) central obesity, as measured using waist circumference, ≥90 cm for men or ≥80 cm for women).

### Procedure and ethical considerations

This study was conducted with a corporate hospital through the community health screening program and approved by the institutional review board ethical committee (Chang-Gung Memorial Hospital Ethics Committee No 102-4399B). Interviewing procedures and privacy protection were explained to the participants by research assistants who were trained for 4 h by the research team, which included the investigators and dentist. To avoid measurement error, eight research assistants were separated into four pairs before the start of the project and were asked to interview an elder in the community activity center, which allowed us to confirm a 90 % correct response rate of inter-rater reliability among these four pairs. Face and content validity of the instrument were judged to be good (0.90–0.92) by a panel of five experts—a faculty member in public health and health education, endocrine and metabolic physicians, and nursing faculty members who teach health promotion. Some items within the instruments were revised according to the experts’ suggestions. Research assistants were all senior nursing students, in an RN-BSN (registered nurse-bachelor of science in nursing) program, had received two consecutive training sessions, each with 2 h duration. In session 1, we focused on understanding research contents and practicing interview skills. In session 2, eight research assistants were grouped into four pairs to become familiar with all items of the questionnaire and the procedures of how to count the number of teeth.

All health examinations were conducted, and venous blood samples were obtained after the participants had fasted for at least 8 h. Serum high-density lipoprotein cholesterol (HDL-C), fasting blood glucose (FBG), and triglyceride (TG) levels were measured enzymatically at the central laboratory of the cooperating hospital. Research assistants measured the height, weight, waist circumference, real teeth, and blood pressure according to standard procedures. Written informed consent was obtained from all participants.

### Statistical analysis

Data are presented as either number (percentage) for categorical variables or mean ± standard deviation (*SD*) for continuous variables. Group comparisons (i.e. by sex or NRT <20) were made using the chi-square test for categorical variables or the independent sample *t*-test for continuous variables. To investigate factors associated with NRT <20, a multivariate logistic regression analysis was conducted. The multivariate analysis was further stratified by sex to examine factors independently associated with NRT <20. All variables were incorporated into the multivariate analyses and considered as confounders. To avoid collinearity among similar parameters (i.e. the three variables for unhealthy dietary habits, the three variables for substance use, and the five components of MetS), we used the general variables (i.e., unhealthy dietary habit, substance use, and component number of MetS) in the multivariate analyses instead [31, 32]. The prevalence of NRT <20 in this study was 16.2 % (1085/6680); this value was low enough to avoid bias and thus warrant the use of logistic regression. A value of *p* <0.05 was considered statistically significant. All data analyses were conducted using SPSS software version 17 (SPSS Inc., Chicago, Illinois).

## Results

### Demographic characteristics

Of the 6823 community adults who participated in this study, 6680 met the criteria and signed the consent form. There were 2901 (43.4 %) male and 3779 (56.6 %) female participants with a mean age of 42.5 years (*SD* = 12.5; Table 1). The mean NRT was 24.6 (*SD* = 7.4), and 1085 (16.3 %) had an NRT <20, including 8.7 % with an NRT of 11–19 and 7.6 % with ≤10. More than half (65.6 %) of the subjects were educated for <12 years, and 57.9 % reported infrequent (never or seldom) use of dental floss. More than 70 % (73.4 %) reported having at least one unhealthy dietary habit or deficient consumption, whereas 27.5 % consumed at least one of the three substances. The most frequent MetS component was high blood pressure (44.2 %), followed by central obesity (36.6 %), high FBG level (28.6 %), low HDL-C level (21.8 %), and high TG level (19.9 %). The mean number of MetS components was 1.5 (*SD* = 1.3), and the prevalence rate of MetS was 24.0 % (Table 1).

**Table 1:** Demographic characteristics and health-related behaviors stratified by gender (*N* = 6680)

Variables	Whole *N* = 6680	Male *N* = 2901	Female *N* = 3779	*P*
NRT (mean ± SD)^a^	24.6 ± 7.4	25.4 ± 6.9	24.1 ± 7.8	<0.001
Group of NRT				
~ 10	506 (7.6)	180 (6.2)	326 (8.6)	<0.001
11 ~ 19	579 (8.7)	207 (7.1)	372 (9.8)	
20 ~ 29	3889 (58.2)	1690 (58.3)	2199 (58.2)	
30~	1706 (25.5)	824 (28.4)	882 (23.3)	
Age, year (mean ± SD)	42.5 ± 12.5	42.4 ± 12.4	42.6 ± 12.6	0.392
20–40	3236 (48.4)	1398 (48.2)	1838 (48.6)	0.717
41–64	3444 (51.6)	1503 (51.8)	1941 (51.4)	
Education level				0.101
≦ Junior high school	4381 (65.6)	1871 (64.5)	2510 (66.4)	
≧ Senior high school	2299 (34.4)	1030 (35.5)	1269 (33.6)	
Using dental floss				<0.001
Infrequent (never/seldom)	3870 (57.9)	1768 (60.9)	2102 (55.6)	
Frequent (often/always)	2810 (42.1)	1133 (39.1)	1677 (44.4)	
Unhealthy dietary habit	4901 (73.4)	2213 (76.3)	2688 (71.1)	<0.001
Deficient of 5 groups	1457 (21.8)	684 (23.6)	773 (20.5)	0.002
Deficient of vegetable	2363 (35.4)	1212 (41.8)	1151 (30.5)	<0.001
Deficient of water	2443 (36.6)	876 (30.2)	1567 (41.5)	<0.001
Substance use	1836 (27.5)	1533 (52.8)	303 (8.0)	<0.001
Alcohol drinking	904 (13.5)	781 (26.9)	123 (3.3)	<0.001
Betel nut chewing	648 (9.7)	617 (21.3)	31 (0.8)	<0.001
Cigarette smoking	1443 (21.6)	1236 (42.6)	207 (5.5)	<0.001
Metabolic syndrome (MetS)^b^	1603 (24.0)	855 (29.5)	748 (19.8)	<0.001
SBP/DBP≧ 130/85 mmHg^c^	2953 (44.2)	1608 (55.4)	1345 (35.6)	<0.001
WC≧90/80 cm (male/female)^d^	2448 (36.6)	1071 (36.9)	1377 (36.4)	0.686
FBG (≧100 mg/dl)^e^	1911 (28.6)	1000 (34.5)	911 (24.1)	<0.001
HDL-C< 40/ 50 mg/dl^f^	1457 (21.8)	573 (19.8)	884 (23.4)	<0.001
Triglyceride(≧150 mg/dl)	1326 (19.9)	864 (29.8)	462 (12.2)	<0.001
Number of MetS component	1.5 ± 1.3	1.8 ± 1.3	1.3 ± 1.3	<0.001

^a^NRT: number of remaining teeth

^b^MetS: ≧3 of 5 components

^c^SBP/DBP: systolic/diastolic blood pressure

^d^WC: waist circumference

^e^FBG: fasting blood glucose

^f^HDL-C: high-density lipoprotein cholesterol, male <40 mg/dl, female <50 mg/dl

Women had fewer mean NRT (24.1 ± 7.8) and a higher percentage of NRT <20 than men (*p* <0.001). Men had less frequent use of dental floss, a higher proportion of deficiency in all dietary habits (except for water consumption), and more substance use compared to women (*p* <0.001). Men also had higher MetS prevalence (29.5 % vs. 19.8 %), blood pressure, FBG level, and TG level and lower prevalence of HDL-C (*p* <0.001). No sex difference was found in the prevalence of central obesity (Table 1).

Subjects with NRT <20 tended to be older, women, and less educated, with less frequent dental floss use, betel nut chewing, and substance use (*p* <0.05) compared to those with NRT ≥ 20. The prevalence of each MetS component, including high systolic blood pressure/diastolic blood pressure, high waist circumference, high FBG level, low HDL-C level, and high TG level, as well as MetS (p <0.05) was significantly higher in subjects with NRT <20 than in those with NRT ≥ 20 (Table 2).

**Table 2:** Factors associated with number of remaining teeth <20

	Whole			Male			Female		
Variable	NRT≧20 (*n* = 5595)	NRT <20 (*n* = 1085)	*P*	NRT≧20 (*n* = 2514)	NRT <20 (*n* = 387)	*P*	NRT≧20 (*n* = 3081)	NRT <20 (*n* = 698)	*P*
Age >40 years	2517 (45.0)	927 (85.4)	<0.001	1177 (46.8)	326 (84.2)	<0.001	1340 (43.5)	601 (86.1)	<0.001
Female gender	3081 (55.1)	698 (64.3)	<0.001	–	–	–	–	–	–
Education < high school	3401 (60.8)	980 (90.3)	<0.001	1532 (60.9)	339 (87.6)	<0.001	1033 (33.5)	536 (76.8)	<0.001
Infrequent dental floss	3061 (54.7)	809 (74.6)	<0.001	1460 (58.1)	308 (79.6)	<0.001	1601 (52.0)	501 (71.8)	<0.001
Unhealthy dietary habit	4102 (73.3)	799 (73.6)	0.825	1914 (76.1)	299 (77.3)	0.627	2188 (71.0)	500 (71.6)	0.745
Deficient of 5 groups	1240 (22.2)	217 (20.0)	0.114	598 (23.8)	86 (22.2)	0.500	642 (20.8)	131 (18.8)	0.221
Deficient of vegetable	1998 (35.7)	365 (33.6)	0.192	1041 (41.4)	171 (44.2)	0.302	957 (31.1)	194 (27.8)	0.090
Deficient of water	2042 (36.5)	401 (37.0)	0.773	764 (30.4)	112 (28.9)	0.563	1278 (41.5)	289 (41.4)	0.971
Substance use	1503 (26.9)	333 (30.7)	0.010	1270 (50.5)	263 (68.0)	<0.001	233 (7.6)	70 (10.0)	0.030
Alcohol drinking	747 (13.4)	157 (14.5)	0.324	656 (26.1)	125 (32.3)	0.010	91 (3.0)	32 (4.6)	0.028
Betel nut chewing	523 ( 9.3)	125 (11.5)	0.027	507 (20.2)	110 (28.4)	<0.001	16 (0.5)	15 (2.1)	<0.001
Cigarette smoking	1191 (21.3)	252 (23.2)	0.156	1021 (40.6)	215 (55.6)	<0.001	170 (5.5)	37 (5.3)	0.820
Metabolic syndrome^a^	1221 (21.8)	382 (35.2)	<0.001	699 (27.8)	156 (40.3)	<0.001	522 (16.9)	226 (32.4)	<0.001
SBP/DBP≧130/85^b^	2347 (41.9)	606 (55.9)	<0.001	1367 (54.4)	241 (62.3)	0.004	980 (31.8)	365 (52.3)	<0.001
WC≧90/80 cm (M/F)^c^	1926 (34.4)	522 (48.1)	<0.001	901 (35.8)	170 (43.9)	0.002	1025 (33.3)	352 (50.4)	<0.001
FBG (≧100 mg/dl)^d^	1449 (25.9)	462 (42.6)	<0.001	812 (32.3)	188 (48.6)	<0.001	637 (20.7)	274 (39.3)	<0.001
HDL-C<40/ 50 mg/dl^e^	1156 (20.7)	301 (27.7)	<0.001	472 (18.8)	101 (26.1)	0.001	684 (22.2)	200 (28.7)	<0.001
TG(≧150 mg/dl)^f^	1077 (19.2)	249 (22.9)	0.005	733 (29.2)	131 (33.9)	0.060	344 (11.2)	118 (16.9)	<0.001
Number of components	1.4 ± 1.3	2.0 ± 1.4	<0.001	1.7 ± 1.3	2.1 ± 1.3	<0.001	1.2 ± 1.3	1.9 ± 1.4	<0.001

*NRT* number of remaining teeth

^a^Metabolic syndrome: three of five items

^b^Systolic blood pressure/diastolic blood pressure

^c^Waist circumference, (male/female≧90/80 cm)

^d^Fasting blood glucose

^e^High-density lipoprotein cholesterol

^f^Triglyceride

After adjusting for confounding factors, multivariate logistic regression showed that age >40 years (odds ratio [OR] = 4.56, 95 % confidence interval [CI]: 3.74–5.55), female sex (OR = 1.88, 95 % CI: 1.56–2.25), less education (OR = 2.4, 95 % CI: 1.90–3.02), infrequent use of dental floss (OR = 1.94, 95 % CI: 1.66–2.27), substance use (OR = 1.32, 95 % CI: 1.09–1.59), and a greater number of MetS components (OR = 1.1, 95 % CI: 1.04–1.16) were independently associated with a higher risk of NRT <20. Unhealthy dietary habits were not independently associated with NRT <20 (Table 3). In further analysis stratified by sex, the results demonstrated that the number of MetS components was not associated with tooth loss for male subjects (OR = 1.04, 95 % CI: 0.96–1.14). In contrast, the association between the number of MetS components and NRT <20 was significant for female subjects (OR = 1.13, 95 % CI: 1.06–1.21). In addition, the OR of the number of MetS components was significantly higher in women than in men, indicating a significant interaction between sex and the number of MetS components (p = 0.026, not shown in the table).

**Table 3:** Multivariate logistic regression of associated factors with number of remaining teeth <20

Predictor	Whole		Male		Female	
	aOR (95 % CI of OR)	*P* value	aOR (95 % CI of OR)	*P* value	aOR (95 % CI of OR)	*P* value
Age (1 > 40 years)	4.56 (3.74–5.55)	<0.001	4.21 (3.10–5.72)	<0.001	4.69 (3.62–6.08)	<0.001
Gender (1 = female)	1.88 (1.56–2.25)	<0.001	–	–	–	–
Education (1 < high school)	2.40 (1.90–3.02)	<0.001	2.14 (1.52–3.01)	<0.001	2.59 (1.89–3.55)	<0.001
Dental floss (1 = infrequent)	1.94 (1.66–2.27)	<0.001	2.23 (1.70–2.92)	<0.001	1.81 (1.49–2.19)	<0.001
Substance use (1 = yes)	1.32 (1.09–1.59)	0.004	1.37 (1.07–1.75)	0.013	1.31 (0.97–1.78)	0.082
Number of MetS component	1.10 (1.04–1.16)	<0.001	1.04 (0.96–1.14)	0.324	1.13 (1.06–1.21)	<0.001
Dietary habit (1 = unhealthy)	1.17 (0.99–1.37)	0.061	1.13 (0.86–1.49)	0.364	1.17 (0.96–1.43)	0.112

*aOR* adjusted odds ratio, *CI* confidence interval, *MetS* metabolic syndrome

## Discussion

If 8020 is the common consensus for pursuing a national health goal, it is necessary to explore the risk factors and possible mechanisms associated with fewer remaining teeth in rural areas. The present findings indicate a high prevalence of NRT <20 and fewer teeth among rural community adults. Despite the unmodifiable factors such as age, sex, and educational level, NRT <20 in rural adults was associated with infrequent use of dental floss, substance use, and MetS. These findings may echo some evidence that oral infection contributes to a low grade systemic inflammatory condition. Consequently, these microbial origins are characterized by loss of tooth attachment, resulting in edentulous if untreated [22]. Further, MetS has an adverse effect on periodontal health and periodontal infection, which further has an adverse effect on glycemic control [26, 27]. In this study, participants aged 20–64 years had a mean of only 24.6 remaining teeth at a mean age of 42.5 years, and 16.3 % had NRT <20. These results seem far from achieving the goal 8020 of Japan and the World Health Organization [1, 2]. Therefore, there is still considerable room for improvement, including how to improve healthy oral hygiene habits and reduce the incidence of MetS among rural adults.

Individuals with age >40 years, female sex, and less education were more prone to have NRT <20. This is similar to the finding of a previous study showing that older age was significantly associated with having 19 or fewer teeth [11]. Although mean age, age distribution, and educational level were similar between both sexes in the present study, women had fewer teeth and a higher percentage of NRT <20 than men. In addition, the association between the number of MetS components and NRT <20 was not significant for men, but was significant for women. Moreover, men had significantly less frequent use of dental floss and more unhealthy dietary habits, substance use, and MetS prevalence than women. Furthermore, there was a significant interaction between sex and factors associated with NRT. The exact reasons are unknown, but may be endocrinological differences between the sexes, such as how estrogen and androgens moderate menopause and differences in bone mineral density. In Japan, Ueno et al. [33] found that higher-parity women are more likely to lose teeth. In conjunction with an old Chinese saying that women “lose one tooth for giving birth to one child,” women may use such findings to justify their tooth loss as reasonable. In a recent study, Darcey et al. [34] reported that osteoporotic patients may be at greater risk of tooth loss. It is necessary to further explore the causal relationship between female sex, bone mineral density, and parity. All individuals, regardless of age or stage of life (such as pregnant or postmenopausal), should have at least 20 functional teeth.

Less educated individuals were at greater risk of NRT <20. This finding is congruent with those of many other studies, indicating that low educational level was related to tooth loss [11–13]. This may be because those with less education likely have lower socioeconomic status and may not possess the knowledge to take care of their teeth or lack easy access to dental services. Although the National Health Insurance of Taiwan, which was launched in 1995 and covers over 99 % of the population, offers oral check-ups twice yearly and aims to provide high quality and affordable health care to all Taiwanese, the limited dental service available has resulted in insufficient oral health service in rural areas [3]. Therefore, there is an urgent need for effective and efficient health policies to enhance accessibility of dental services and to individualize oral health education tailored to the culture.

Evidence supports that adequate use of dental floss after tooth brushing is important to oral hygiene and prevents tooth decay and periodontal disease [22]. More than half of participants reported that they seldom used dental floss after tooth brushing each day. Infrequent use of dental floss was associated with a higher risk of NRT <20 in this study. This result is similar to another study in Taiwan, wherein Hsu et al. [12] reported that those with low dentition were more likely to use dental floss less frequently. Compared with nationwide statistics, participants used dental floss (55.4 vs. 42.1 %) less often than the general population [3]. Three decades ago, dental floss was not even introduced as a routine daily practice in Taiwan, although tooth brushing was a common habit. The current study also found that men had significantly less frequent use of dental floss than women. However, women tended to have fewer teeth than men. Further study is needed to explain this seemingly disparate result. Dental hygiene should include dental floss and daily flossing is recommended [35].

In the present study, 27.5 % of the participants reported using alcohol, betel nuts, or cigarettes. Substance use was significantly associated with a higher risk of NRT <20. These results are consistent with those of previous studies [11, 12, 18]. Therefore, health care providers in primary settings need to educate the public about the influence of substance use (on tooth loss) and initiate a betel nut chewing and smoking cessation program. Additionally, a previous study showed that smoking was a major factor for tooth loss in postmenopausal women [33, 36]. In the present study, women were not asked about their menopausal status. Further study should consider the relationship between tooth loss and menopausal status.

The prevalence of MetS in the present study was high (24.0 %). A greater number of MetS components was independently associated with a higher risk of NRT <20; this is consistent with the findings of previous studies, which showed that higher body mass index [16], high FBG level [37], low HDL-C level, and high TG level were related to the number of missing teeth [5]. These results demonstrate a need for further study to understand more fully the mechanisms underlying the association of MetS with tooth loss.

Based on the present findings, additional actions need to be taken; including the dissemination of research findings to and educating the general public about factors associated with tooth loss, provision of regular dental services, and increased government involvement. A referral health care system could be established and regulated. Newly diagnosed MetS or diabetic patients should be referred for dental care. If a dentist suspects a client may have MetS or diabetes mellitus, referrals should be made for the patient to visit an endocrinologist.

### Limitations

This study has limitations. First, only natural and filled teeth remaining in the oral cavity were counted by the research assistants. A more comprehensive dental examination by a dentist, including a visual evaluation of oral mucous membranes and a digital examination, is difficult to carry out in local hospitals. This might limit the study regarding the causes of tooth loss, such as periodontal disease. Second, the samples were not entirely random, and most of the participants had lower socioeconomic status. This limits any generalization of these findings and may be a potential threat to the internal validity of the health literacy issue, which requires consideration. To better understand factors related to tooth loss, future studies should focus on the following aspects: periodontitis, dental caries, economic status, frequency of tooth brushing, regular dental check-ups, history of diabetes mellitus, bone density, menopause status, and amount or type of substances consumed.

## Conclusion

Despite some limitations, this study showed that a high prevalence of rural adults had NRT <20. Furthermore, despite unmodifiable factor, such as age, sex, and education, NRT <20 in rural adults was associated with unhealthy habits, including infrequent use of dental floss, chewing betel nuts, and MetS. Further research is needed to understand the mechanisms underlying the relationship between MetS and tooth loss, to explore ways to prevent teeth loss, and to enhance cessation of use of specific substances in rural populations.

## Abbreviations

FBG: fasting blood glucose
HDL-C: high-density lipoprotein cholesterol
MetS: metabolic syndrome
NRT: number of remaining teeth
TG: triglyceride
WC: waist circumference
